# From media engagement to global impact: a serial moderated mediation model of cultural relatability and emotional resonance in Chinese culture communication

**DOI:** 10.3389/fpsyg.2026.1782027

**Published:** 2026-03-17

**Authors:** Mengying Xiong, Yijin Chen, Yunqian Zhou

**Affiliations:** 1 School of Journalism and Communication, Nanchang University, Nanchang, China; 2 School of Broadcasting and Hosting Art, Wuhan College of Communication, Wuhan, China

**Keywords:** affective disposition theory, Chinese culture communication, cultural diplomacy, cultural relatability, elaboration likelihood model, emotional resonance, global impact, media engagement

## Abstract

**Introduction:**

Despite substantial research on media influence and cultural communication, the mechanisms through which media engagement drives global impact remain underexplored, particularly within the context of Chinese culture communication. This study aims to fill this gap by examining how media engagement translates into global impact through the serial mediating roles of cultural relatability and emotional resonance, moderated by the effectiveness of Chinese culture communication.

**Methods:**

Grounded in the elaboration likelihood model and affective disposition theory, this study employs a time-lagged quantitative research design, collecting data from 411 university students in China.

**Results:**

The findings reveal significant positive relationships between media engagement, cultural relatability, emotional resonance, and global impact, highlighting the critical mediating roles of cultural relatability and emotional resonance. The study also demonstrates that effective Chinese culture communication amplifies the influence of media engagement on cultural relatability.

**Discussion:**

These insights contribute to existing theoretical frameworks and provide practical implications for media producers, cultural policymakers, and communication strategists aiming to enhance global cultural orientation through strategically designed cultural media content. The study acknowledges its limitations and suggests avenues for future research, advocating for more diverse samples and longitudinal designs to deepen the understanding of media-driven cultural impact.

## Introduction

Media plays a central role in shaping cultural perceptions and facilitating cross-cultural understanding. With the expansion of digital platforms and global connectivity, media has become a strategic instrument for disseminating cultural narratives beyond national borders. Scholars emphasize its influence in constructing social realities and shaping attitudes (Cheng et al., 2024; Trunfio and Rossi, 2021). According to Yang et al. (2024), governments increasingly rely on media engagement as a tool of cultural diplomacy and soft power, particularly in the context of China’s growing global cultural initiatives.

Despite this recognition, research has largely examined media engagement in terms of attention, interaction, or behavioral outcomes (Meng and Leung, 2021; Trunfio and Rossi, 2021; Yang et al., 2024), while macro-level studies of public diplomacy focus on broad international influence (Chan-Olmsted and Wolter, 2018; Gerodimos, 2021; Kurki, 2022; Roberts, 2018). What remains insufficiently understood is how engagement with cultural media content translates into broader cultural perceptions. In particular, the cognitive and emotional mechanisms that connect media engagement to global-level attitudinal outcomes remain underexplored. This gap is especially relevant in Chinese culture communication, where media functions not only as entertainment but as a structured vehicle of cultural representation (Johnson et al., 2023).

To address this limitation, this study conceptualizes media engagement as the starting point of a sequential influence process. Media engagement involves cognitive, emotional, and behavioral involvement with content (Ren et al., 2024), and engaged audiences are more likely to internalize media narratives (Meng and Leung, 2021). However, engagement alone does not explain broader cultural influence. One potential mechanism is cultural relatability—the degree to which media narratives align with audiences’ cultural values and experiences (Atiq et al., 2022; Schwartz et al., 2022). Although cultural relatability has been examined in marketing contexts (Orencia, 2023), its mediating role in cultural communication models remains insufficiently examined.

Beyond cognitive alignment, emotional resonance represents an affective pathway of influence. Emotional resonance refers to the meaningful emotional responses evoked by media content (Gu, 2024; Vorderer and Halfmann, 2019), which strengthen message retention and attitudinal commitment (Bartsch and Viehoff, 2010). Affective disposition theory explains how emotional responses shape audience evaluations and broader perceptions (Zillmann, 2003; Zillmann and Cantor, 2017). Yet few studies integrate emotional resonance into structured frameworks linking engagement to global cultural outcomes.

In this study, global impact is conceptualized as audiences’ global cultural orientation—namely, the extent to which engagement with Chinese cultural media content shapes openness to cultural diversity and perceptions of China within an international context (Chan-Olmsted and Wolter, 2018; Inglehart et al., 2014). Rather than directly measuring foreign audiences, the study examines how domestically situated audiences internalize globally oriented cultural narratives, consistent with scholarship on mediated globalization and cultural diplomacy (Gerodimos, 2021; Kurki, 2022).

Furthermore, the effectiveness of Chinese culture communication may strengthen these processes. Effective cultural communication involves clear and authentic presentation of cultural narratives (Gong et al., 2021), and strategic articulation enhances relatability and engagement (Johnson et al., 2023). Accordingly, this study proposes that Chinese culture communication moderates the relationship between media engagement and cultural relatability. Specifically, anchored in the elaboration likelihood model (Petty et al., 1986; O’Keefe, 2013) and affective disposition theory (Zillmann, 2003), this research develops a serial moderated mediation model linking media engagement to global cultural orientation through cultural relatability and emotional resonance. By clarifying these sequential mechanisms, the study addresses a critical gap in understanding how media engagement contributes to global cultural impact within the framework of Chinese culture communication.

## Theoretical background

### A cognitive–affective framework of media-driven cultural influence

The study postulates that understanding how media engagement translates into broader cultural influence requires a framework capable of explaining both cognitive processing and emotional response. While many communications theories address persuasion or affect independently, fewer integrate these dimensions within a sequential mechanism of influence. To address this gap, the present study integrates the elaboration likelihood model (ELM; Petty et al., 1986; O’Keefe, 2013) and affective disposition theory (ADT; Zillmann, 2003) to construct a cognitive–affective framework explaining how engagement-driven processing contributes to global cultural orientation.

The ELM explains how individuals process persuasive messages through either central or peripheral routes (Petty et al., 1986). Central route processing involves active cognitive elaboration that produces more stable and enduring attitude change, whereas peripheral processing relies on superficial cues and generates more temporary effects (Shahab et al., 2021). Within media engagement contexts, higher levels of engagement increase the likelihood of central processing (Li et al., 2021; Moradi and Zihagh, 2022). In cultural communication settings, such deep processing strengthens evaluative alignment with cultural narratives, enhancing perceived cultural relatability (Orencia, 2023; Reade, 2021). However, although ELM effectively explains cognitive evaluation and persuasion, it does not fully account for how these cognitive appraisals evolve into emotionally grounded dispositions that shape broader cultural perceptions.

On the other hand, ADT complements this limitation by focusing on emotional responses as drivers of attitudinal outcomes (Zillmann, 2003). According to ADT, individuals develop affective dispositions toward media content based on emotional reactions, which subsequently guide interpretation and evaluative judgment (Zillmann and Cantor, 2017). Emotional resonance enhances empathy, identification, and message acceptance (Bartsch and Viehoff, 2010), and has been shown to strengthen cultural connection and audience engagement (Mühlhoff, 2019). Yet ADT alone does not specify the cognitive antecedents that give rise to these emotional states.

The integration of ELM and ADT is therefore analytically necessary rather than merely complementary. ELM clarifies how engagement activates central cognitive processing and fosters cultural evaluation, whereas ADT explains how these cognitive evaluations generate emotional resonance that amplifies attitudinal influence. Together, they form a sequential mechanism in which engagement facilitates cognitive alignment, cognitive alignment fosters emotional resonance, and emotional resonance contributes to global cultural orientation. Employing either framework independently would yield a partial explanation: ELM would account for persuasion without affective amplification (Petty et al., 1986), whereas ADT would explain emotional influence without clarifying its cognitive origins (Zillmann, 2003). By integrating these perspectives, the present study advances a unified cognitive–affective model that captures the layered dynamics of media-driven cultural impact.

**Figure 1:**
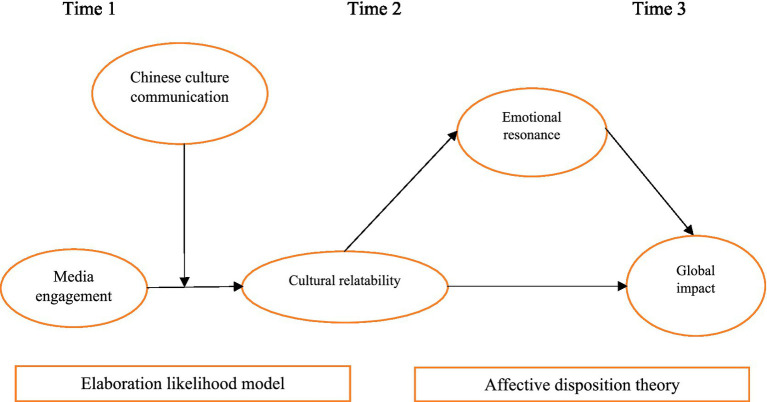
Conceptual model.

Last but not the least, this integration is particularly appropriate in the context of Chinese culture communication, where both rational evaluation of cultural narratives and emotional identification with them shape broader international perception (Johnson et al., 2023; Gong et al., 2021). By positioning cultural relatability as a cognitive mediator grounded in ELM and emotional resonance as an affective mediator grounded in ADT, the model offers added explanatory value beyond single-theory applications and clarifies the distinct theoretical contribution of the study (see Figure 1).

### Linking media engagement with cultural relatability

Building on the conceptualization introduced earlier, media engagement reflects the degree to which audiences cognitively and emotionally involve themselves with media content (Ren et al., 2024). High media engagement often leads to increased attention, immersion, and a stronger personal connection with the content, which can influence attitudes, beliefs, and behaviors (Bartsch and Viehoff, 2010). Previous research highlights that when audiences are engaged with media content, they are more likely to internalize its messages, contributing to lasting attitudinal and behavioral changes (Meng and Leung, 2021; Yang et al., 2024). In cultural communication contexts, such engagement increases the likelihood that audiences will actively interpret cultural narratives rather than passively consume them.

Cultural relatability, on the other hand, refers to the extent to which media content aligns with the cultural experiences, values, and beliefs of its audience (Reade, 2021). When media content reflects familiar cultural elements, it creates a sense of validation and comfort, enhancing the likelihood of message acceptance (Atiq et al., 2022). Studies have shown that culturally relatable content fosters both cognitive and emotional connections, enabling audiences to process information more deeply and respond more positively (Seidel et al., 2023). Cultural relatability not only influences individual attitudes but also contributes to broader societal impacts by promoting cultural understanding and reducing cultural biases.

According to ELM, deeply engaged audiences are more likely to process media content through the central route, where they critically evaluate and relate to the content on a cognitive level (Madadi et al., 2024). This central processing enhances cultural relatability by allowing audiences to draw connections between media messages and their own cultural frameworks (Mardhatilah et al., 2023). Previous studies support this theoretical inference by demonstrating how engaged media audiences develop stronger cultural connections. For example, Schwartz et al. (2022) found that media engagement enhances cultural relatability by promoting deeper cognitive processing of content. Similarly, Atiq et al. (2022) showed that culturally relatable media content positively influences how audiences perceive and respond to cultural messages. Tam et al. (2022) also identified that media engagement plays a pivotal role in bridging cultural gaps through relatable content. Moreover, Ahmed et al. (2024) highlighted the emotional aspect, noting that engaged audiences not only process cultural content more thoroughly but also experience stronger emotional connections. These findings align with Abbas et al. (2021), who emphasized that culturally rich media content supports cultural diplomacy by fostering global cultural understanding.

Building on these insights, this study hypothesizes that media engagement positively influences cultural relatability. The proposed hypothesis is:

*H1*: Media engagement has a positive effect on cultural relatability.

### Linking cultural relatability with emotional resonance

Emotional resonance refers to the extent to which media content evokes strong, meaningful emotional responses from its audience (Bartsch and Viehoff, 2010). Emotional resonance is critical in media communication as it enhances message retention, increases the likelihood of sharing content, and fosters a stronger bond between the audience and the media source (Gu, 2024). When media content resonates emotionally, it often leads to more profound attitudinal and behavioral impacts, suggesting that emotions play a pivotal role in how media influences individuals and broader cultural perceptions.

A closer look at the relationship between cultural relatability and emotional resonance reveals an intrinsic connection between these constructs. When media content is culturally relatable, it naturally sets the stage for emotional resonance by offering audiences a sense of familiarity and validation (Rigby and Lee, 2024). Culturally relatable content aligns with audiences’ lived experiences, making the media messages more authentic and trustworthy, which in turn triggers deeper emotional responses (Kim and Drumwright, 2016). Unlike generic media messages, culturally tailored content can evoke specific emotions such as pride, nostalgia, or empathy, enhancing the overall impact of the media (Lee et al., 2022).

From theoretical standpoint, ELM suggests that culturally relatable content facilitates central route processing, where audiences engage cognitively with the content, leading to stronger emotional outcomes (Petty et al., 1986). Simultaneously, ADT posits that positive emotional dispositions are more likely to form when audiences connect culturally with media content, resulting in heightened emotional resonance (Hinsz et al., 2019). This theoretical perspective indicates that cultural relatability is not merely a cognitive construct but also a precursor to significant emotional experiences. When audiences relate to the cultural aspects of media content, they are more likely to develop an emotional bond with the content, enhancing the media’s influence on attitudes and behaviors.

Building upon these insights, the study proposes the following hypotheses to empirically test these theoretical assumptions:

*H2*: Cultural relatability has a positive effect on emotional resonance.

*H3*: Cultural relatability mediates the relationship between media engagement and emotional resonance.

### Linking emotional resonance with global impact

As clarified earlier, global impact is conceptualized in this study as audiences’ global cultural orientation, reflecting openness to cultural diversity and perceptions of Chinese culture within an international framework (Inglehart et al., 2014). Media content that achieves a global impact often transcends cultural boundaries, promoting understanding, tolerance, and shared values among diverse audiences (Chen and Starosta, 2008). When media content successfully influences global impact, it plays a strategic role in enhancing a nation’s soft power by cultivating a positive image and fostering stronger international ties.

The link between emotional resonance and global impact is well-supported in media studies. When audiences experience strong emotional responses to media content, they are more likely to internalize the messages, share the content with others, and engage in behaviors aligned with the intended message (Bartsch and Viehoff, 2010). Emotions act as a catalyst, transforming passive media consumption into active engagement, where audiences become advocates of the cultural narratives presented (Lee, 2019). For instance, Wang et al. (2023) observed that emotionally resonant cultural media content not only enhances immediate audience engagement but also leads to longer-term changes in attitudes towards the represented culture. This aligns with findings from Thomas (2024), who highlighted that emotional resonance enhances the global reach of cultural messages by making them more memorable and impactful.

From a theoretical perspective, ELM posits that when media content engages audiences emotionally, it facilitates deeper processing through the central route, leading to more durable attitudinal changes (Petty et al., 1986). Emotional resonance, by enhancing the depth of message processing, increases the likelihood that the media’s cultural and ideological messages will contribute to a broader global impact (Kanchan and Gaidhane, 2023). ADT further supports this notion by suggesting that positive emotional responses enhance audiences’ dispositions toward the media content, promoting cultural openness and acceptance (Zillmann, 2003). Considering these theoretical and empirical insights, this study proposes the following hypotheses to test the role of emotional resonance in achieving global impact:

*H4*: Emotional resonance has a positive effect on global impact.

*H5*: Emotional resonance mediates the relationship between cultural relatability and global impact.

### A serial mediation model

Building on the preceding arguments, the proposed model assumes a sequential pathway in which media engagement enhances cultural relatability, which subsequently fosters emotional resonance, ultimately contributing to global cultural orientation (Hayes, 2018; Preacher and Hayes, 2008). Rather than treating these mechanisms as independent processes, the model conceptualizes them as interdependent stages within a structured cognitive–affective progression. This structure reflects the cognitive–affective sequence outlined by ELM (Petty et al., 1986) and ADT (Zillmann, 2003), where central processing facilitates evaluative alignment and affective responses translate this alignment into broader attitudinal outcomes. Prior research supports the usefulness of serial mediation in capturing layered communication effects (Zhuang et al., 2023), particularly in contexts where persuasion unfolds through multiple psychological stages.

*H6*: Media engagement has a positive effect on global impact through the serial mediation of cultural relatability and emotional resonance.

### Moderating role of Chinese culture communication

Chinese culture communication refers to the strategic dissemination of Chinese cultural values, traditions, and narratives through media and other communication channels (Chen and Starosta, 2008). Effective cultural communication involves presenting authentic, clear, and engaging messages that resonate within an international communication context and promote a positive perception of Chinese culture (Gong et al., 2021). Scholars argue that well-communicated cultural messages can bridge cultural gaps, reduce stereotypes, and facilitate cultural exchange (Leng et al., 2021).

When Chinese culture communication is executed effectively, it enhances the influence of media engagement by reinforcing cultural relatability and amplifying emotional resonance (Liu et al., 2022). For example, media content that accurately portrays Chinese traditions and values not only fosters cultural understanding but also strengthens the cognitive and emotional impact of the media messages (Bartsch and Viehoff, 2010). Moreover, the effectiveness of Chinese culture communication can act as a moderating factor, influencing the strength of the relationship between media engagement and cultural relatability (Fang, 2014). Previous studies have demonstrated that cultural communication strategies significantly impact how audiences process and respond to media content, suggesting that a strong cultural communication strategy can enhance media’s role in cultural diplomacy and international relations (Chen and Starosta, 2008; Inglehart et al., 2014).

Conceptually, Chinese culture communication operates as a contextual amplifier. When cultural narratives are communicated with clarity and authenticity, they provide stronger cognitive cues that facilitate central route processing (Zhou et al., 2016), thereby strengthening the linkage between engagement and perceived cultural alignment. Additionally, ADT supports the idea that effective cultural communication creates favorable emotional dispositions toward the content, enhancing its overall impact (Talhelm et al., 2023). Empirical studies also indicate that moderated relationships in media communication are often strengthened when cultural narratives are communicated with clarity and authenticity, highlighting the critical role of Chinese culture communication as a strategic tool in achieving global cultural influence (Dai and Wang, 2024). Based on these insights, the study proposes the following hypothesis:

*H7*: Chinese culture communication moderates the relationship between media engagement and cultural relatability, such that the relationship is stronger when Chinese culture communication is high.

## Methodology

The study employs a quantitative research design utilizing a time-lagged data collection approach to investigate the serial moderated mediation model of media engagement, cultural relatability, emotional resonance, and global impact within the context of Chinese culture communication. The study targets university students as the primary sample due to their accessibility, stability, and relevance to the research objectives. Participants were required to meet the following inclusion criteria: (1) current enrollment in a Chinese university, (2) regular exposure to media content related to cultural communication, and (3) willingness to participate across three survey waves. Students who did not complete all three waves or provided incomplete responses were excluded from the final analysis. This approach aligns with prior studies suggesting that university settings offer an ideal environment for longitudinal research, allowing for consistent face-to-face interactions and ensuring participant retention over multiple time points (e.g., Podsakoff et al., 2003).

A convenience sampling method is utilized, engaging participants from relevant academic programs such as media studies, cultural studies, and international relations to ensure that the sample is familiar with media content and capable of providing informed insights. Recruitment was conducted through classroom announcements and coordination with program instructors, who permitted data collection during scheduled sessions. Participation was voluntary, and no academic incentives were provided. Approximately 500 students participate in the initial data collection phase, with efforts made to retain the same participants throughout the study’s duration to maintain the integrity of the longitudinal design. The first wave yields a high response rate (467 responses), while the second wave experiences a slight drop-off (431 responses), and the third wave achieves a final sample size of 411 completed responses. Cases with patterned responses or excessive missing data were removed prior to final analysis to ensure data quality.

The study is conducted over three phases, with data collection occurring at three distinct time points to capture the sequential relationships between media engagement, cultural relatability, emotional resonance, and global impact. The first wave (time 1) assesses media engagement and Chinese culture communication, the second wave (time 2) measures cultural relatability and emotional resonance, and the third wave (time 3) evaluates global impact. Following Podsakoff et al. (2003), a time lag of approximately 4 weeks is maintained between each data collection phase to mitigate common method bias and allow for the temporal separation of variables. This methodological approach enables a robust examination of both mediation and moderation effects while accounting for potential changes in participants’ perceptions and attitudes over time.

All participants receive a detailed cover letter explaining the study’s objectives, data confidentiality, and the voluntary nature of participation. Ethical considerations are strictly adhered to, ensuring informed consent, the right to withdraw at any stage, and the anonymity of responses. The study obtains ethical clearance from the relevant institutional review board prior to data collection. During face-to-face sessions, participants are guided through the questionnaire, and any queries are addressed to ensure data quality. Measures are also taken to minimize social desirability bias by emphasizing the importance of honest and reflective responses.

Demographic data is collected during the initial phase to understand the sample’s characteristics. The sample includes 53 percent females and 47 percent males, with the majority of participants aged between 18 and 22 years (61 percent). A smaller proportion of participants are aged 23 to 25 years (19 percent), while 20 percent are above 25 years of age. The academic background of participants primarily includes media studies (27 percent), cultural studies (23 percent), international relations (19 percent), and other relevant disciplines (31 percent).

Given that the study examines attitudinal and perceptual constructs related to media engagement and cultural orientation, university students constitute a theoretically relevant population, as they represent active media consumers and are frequently engaged in discussions surrounding cultural narratives and international perspectives. While the use of convenience sampling limits broad generalizability, the time-lagged design, structured eligibility criteria, and high retention rate strengthen the internal validity of the findings.

### Measures

The study utilizes established measurement scales to assess media engagement, cultural relatability, emotional resonance, global impact, and Chinese culture communication, ensuring reliability and validity through previously validated instruments. Media engagement is measured using a four-item scale adapted from Calder et al. (2009), capturing participants’ attention, interaction, and emotional involvement with media content. Cultural relatability is assessed with a three-item scale informed by the work of Kim and Gudykunst (2005), focusing on the degree to which individuals perceive media content as reflective of their own cultural experiences. Emotional resonance is measured through a three-item scale adapted from Bartsch and Viehoff (2010), evaluating the emotional responses elicited by the media content. Global impact is assessed using a three-item scale from Inglehart et al. (2014), examining how media exposure influences participants’ global perspectives and attitudes toward Chinese culture. Chinese culture communication is evaluated using a seven-item scale based on the framework provided by Chen and Starosta (2008), capturing the effectiveness, clarity, and authenticity of Chinese cultural messages conveyed through media. All items are rated on a five-point Likert scale ranging from “strongly disagree” to “strongly agree,” with higher scores indicating greater agreement with the constructs. These measurement scales are chosen based on their robust psychometric properties and alignment with the study’s conceptual framework, providing a solid foundation for the structural equation modeling analysis (see Appendix 1).

### Analytical strategy

The study employs partial least squares structural equation modeling (PLS-SEM) to analyze the proposed serial moderated mediation model, examining the relationships between media engagement, cultural relatability, emotional resonance, global impact, and the moderating effect of Chinese culture communication. PLS-SEM is chosen over covariance-based structural equation modeling (CB-SEM) due to its suitability for exploratory research, predictive modeling, and complex models with multiple mediators and a moderator (Hair et al., 2019). Unlike CB-SEM, which focuses on model fit and theory confirmation, PLS-SEM is particularly effective when the research objective is to maximize the explained variance (R^2^) of the dependent variables and when the data do not meet the stringent normality assumptions required by CB-SEM (Sarstedt et al., 2021). Additionally, PLS-SEM handles smaller sample sizes more robustly, which is beneficial considering the longitudinal nature of this study and the reduced sample size at the third wave (411 responses). This approach aligns with contemporary methodological guidelines, emphasizing its strength in evaluating complex mediation and moderation effects within a serial framework (Hair et al., 2021).

## Results

### Measurement model

In the measurement model, it is essential to analyze convergent validity, discriminant validity, and reliability of the constructs. Convergent validity is assessed through outer loadings (ideally >0.70) and average variance extracted (AVE) (>0.50) (Hair et al., 2019). Discriminant validity is evaluated using the Fornell-Larcker criterion, heterotrait-monotrait (HTMT) ratio, and ensuring that each construct’s square root of AVE is higher than its correlations with other constructs (Fornell and Larcker, 1981). Composite reliability and Cronbach’s alpha are examined to ensure internal consistency, with values above 0.70 indicating good reliability (Sarstedt et al., 2021). These analyses ensure that the constructs are both distinct and well-measured, providing a robust foundation for the structural model assessment.

Table 1 presents the outer loadings of measurement items for each construct, including Chinese culture communication, cultural relatability, emotional resonance, global impact, and media engagement. Outer loadings reflect the extent to which each item contributes to its respective construct, with loadings above 0.70 generally considered acceptable for establishing convergent validity (Hair et al., 2019). Most items demonstrate strong outer loadings, particularly within the constructs of cultural relatability (ranging from 0.829 to 0.890), emotional resonance (0.773 to 0.907), global impact (0.783 to 0.842), and media engagement (0.738 to 0.889), indicating a high degree of item reliability. While a few items within the Chinese culture communication construct show loadings slightly below the recommended 0.70 threshold (e.g., 0.622 and 0.666), these values remain within an acceptable range for exploratory research, as outer loadings between 0.60 and 0.70 can still be retained if overall construct reliability is strong (Sarstedt et al., 2021). The high loadings across most items suggest that the measurement model exhibits robust convergent validity, supporting the use of these constructs in subsequent structural model analysis.

**Table 1:** Outer loadings.

Indicators	CCC	CREL	ERES	GIMP	MENG
CCC1	0.622				
CCC2	0.666				
CCC3	0.756				
CCC4	0.752				
CCC5	0.656				
CCC6	0.742				
CCC7	0.757				
CREL1		0.890			
CREL2		0.890			
CREL3		0.829			
ERES1			0.773		
ERES2			0.887		
ERES3			0.907		
GIMP1				0.842	
GIMP2				0.835	
GIMP3				0.783	
MENG1					0.772
MENG2					0.738
MENG3					0.889
MENG4					0.814

MENG: media engagement; CREL: cultural relatability; ERES: emotional resonance; GIMP: global impact; CCC: Chinese culture communication.

Table 2 presents the construct reliability and validity for Chinese culture communication, cultural relatability, emotional resonance, global impact, and media engagement. The Cronbach’s alpha values range from 0.758 to 0.839, indicating satisfactory internal consistency, as values above 0.70 are considered acceptable (Hair et al., 2019). Composite reliability (rho_c) values exceed the 0.70 threshold for all constructs, demonstrating strong construct reliability. The average variance extracted (AVE) values range from 0.503 to 0.757, with most constructs surpassing the recommended 0.50 level, confirming convergent validity (Fornell and Larcker, 1981). Although the AVE for Chinese culture communication is slightly above the minimum criterion (0.503), it is still acceptable for exploratory research. Overall, the results indicate that the measurement model possesses adequate reliability and validity, supporting its suitability for further structural model analysis.

**Table 2:** Construct reliability and validity.

Constructs	Cronbach’s alpha	Composite reliability (rho_a)	Composite reliability (rho_c)	Average variance extracted (AVE)
CCC	0.838	0.844	0.876	0.503
CREL	0.839	0.842	0.903	0.757
ERES	0.819	0.841	0.893	0.735
GIMP	0.758	0.769	0.861	0.673
MENG	0.820	0.839	0.880	0.649

MENG: media engagement; CREL: cultural relatability; ERES: emotional resonance; GIMP: global impact; CCC: Chinese culture communication.

Table 3 presents the discriminant validity of the constructs using the heterotrait-monotrait (HTMT) ratio. Discriminant validity is established when the HTMT values are below the recommended threshold of 0.85 (Henseler et al., 2015). All HTMT ratios in this study fall within the acceptable range, indicating that each construct is sufficiently distinct from the others. The highest HTMT value is 0.842 between emotional resonance and media engagement, which is close to the threshold but still within an acceptable limit, suggesting that while these constructs are related, they maintain discriminant validity. The results affirm that the constructs measure unique concepts and do not exhibit excessive overlap, supporting the robustness of the measurement model for further structural analysis.

**Table 3:** Discriminant validity using HTMT ratio.

Constructs	CCC	CREL	ERES	GIMP	MENG
CCC					
CREL	0.532				
ERES	0.597	0.394			
GIMP	0.356	0.752	0.603		
MENG	0.399	0.374	0.842	0.619	

MENG: media engagement; CREL: cultural relatability; ERES: emotional resonance; GIMP: global impact; CCC: Chinese culture communication.

**Figure 2:**
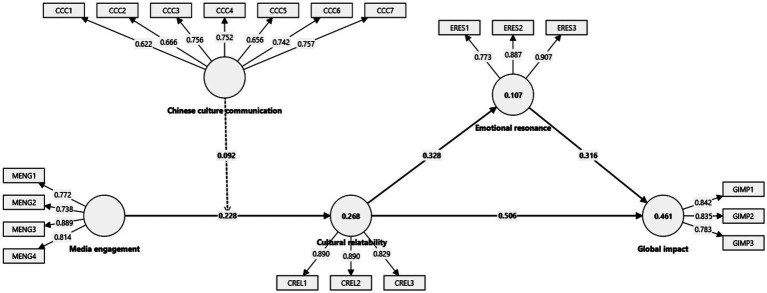
Structural equation model.

Table 4 presents the effects of hypothesized relationships on endogenous variables, along with the impact of control variables including age, gender, and education level. Among the control variables, only gender demonstrates a statistically significant effect (*β* = 0.098, *p* < 0.05), indicating that gender differences may influence outcomes within the model. In contrast, age (*β* = 0.050, n.s.) and education level (*β* = 0.049, n.s.) do not show significant relationships with the dependent variables, suggesting that these demographic factors do not substantially affect the constructs under study. The hypothesis testing reveals strong support for the proposed relationships, with media engagement positively impacting cultural relatability (*β* = 0.228, *p* < 0.05), highlighting how increased engagement with media enhances individuals’ perception of cultural relatability. The relationship between cultural relatability and emotional resonance is also significant (*β* = 0.328, *p* < 0.05), underscoring the role of cultural connections in fostering emotional responses. Furthermore, emotional resonance significantly influences global impact (*β* = 0.316, *p* < 0.05), suggesting that strong emotional connections can drive broader outcomes, potentially enhancing the influence of Chinese culture on a global scale (Figure 2).

**Table 4:** Effects on endogenous variables.

Hypotheses	*β*	CI (5, 95%)	STDEV	*t*-value	*p*-value	Decision	*f^2^*	*R^2^*	*Q^2^*
Age^1^	0.050 (*n.s.*)	(−0.041, 0.097)	0.20	0.250	0.754				
Gender^2^	0.098**	(−0.028, 0.169)	0.03	3.267	0.000				
Education level^3^	0.049 (*n.s.*)	(−0.032, 0.078)	0.20	0.245	0.699				
*H1* MENG → CREL	0.228***	(0., 0.332)	0.053	4.217	0.000	Supported	0.148	0.268	0.419
*H2* CREL → ERES	0.328***	(0.245, 0.413)	0.051	6.372	0.000	Supported	0.483	0.107	0.109
*H4* ERES → GIMP	0.316***	(0.229, 0.401)	0.052	6.058	0.000	Supported	0.195	0.461	0.280
*H7* CCC x MENG → CREL	0.092*	(0.005, 0.189)	0.056	1.650	0.099	Supported	0.239		

MENG: media engagement; CREL: cultural relatability; ERES: emotional resonance; GIMP: global impact; CCC: Chinese culture communication; *** significance *p* < 0.05 (*t* > 1.96); * significance *p* < 0.10 (*t* > 1.65). ^1,2,3^ = control variables.

**Figure 3:**
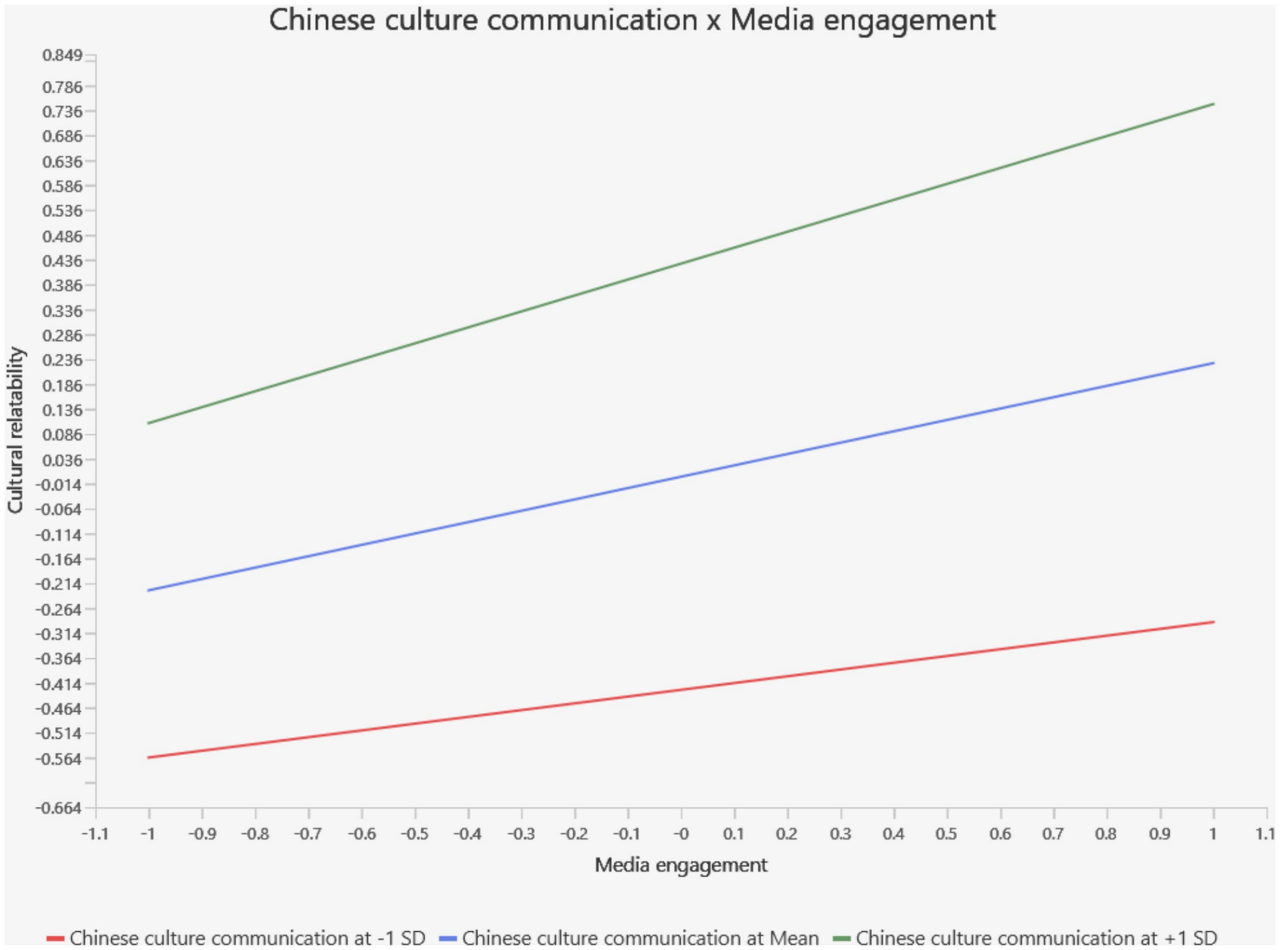
Interaction effect of media engagement and Chinese culture communication.

Additionally, the moderating effect of Chinese culture communication on the relationship between media engagement and cultural relatability is supported (*β* = 0.092, *p* < 0.10), indicating that effective communication of Chinese culture can strengthen the positive influence of media engagement on perceived cultural relatability (Figure 3). The effect sizes (*f*^2^) range from moderate to strong, particularly for the impact of cultural relatability on emotional resonance (*f*^2^ = 0.483), which demonstrates a substantial effect. The *R*^2^ values reveal that the model explains 26.8% of the variance in cultural relatability, 10.7% in emotional resonance, and 46.1% in global impact, indicating a reasonably strong explanatory power, especially for the ultimate outcome of global impact. The predictive relevance (*Q*^2^) values, all above zero, further validate the model’s predictive strength, demonstrating that the constructs not only fit well within the proposed theoretical framework but also contribute meaningfully to understanding how media engagement, through cultural relatability and emotional resonance, can lead to a measurable global impact.

Table 5 presents the results of the mediating effect tests, examining the indirect paths within the proposed serial moderated mediation model. All tested mediation pathways demonstrate statistically significant indirect effects, with t-values exceeding the critical threshold of 1.96 (*p* < 0.05). The first indirect path (H3) from media engagement through cultural relatability to emotional resonance (*β* = 0.075, *t* = 3.260, 95% BCCI [0.042, 0.116]) highlights that higher media engagement enhances cultural relatability, which in turn increases emotional resonance. The second indirect effect (H5) from cultural relatability through emotional resonance to global impact (*β* = 0.103, *t* = 3.802, 95% BCCI [0.064, 0.153]) suggests that when cultural relatability evokes strong emotional responses, it positively influences the global impact of Chinese culture communication. The third and most comprehensive mediation pathway (H6) from media engagement through cultural relatability and emotional resonance to global impact (*β* = 0.024, *t* = 2.726, 95% BCCI [0.012, 0.040]) demonstrates the full serial mediation effect, indicating that the proposed model effectively captures the sequential influence of media engagement on global impact through the dual mediators of cultural relatability and emotional resonance. These results provide robust empirical support for the mediating roles of cultural relatability and emotional resonance in translating media engagement into a significant global impact, emphasizing the nuanced processes through which media strategies can influence broader cultural perceptions.

**Table 5:** Summary of mediating effect tests.

Relationships	Path	*t*-value	95% BCCI
Indirect effect			
*H3* MENG → CREL → ERES	0.075***	3.260	(0.042, 0.116)
*H5* CREL → ERES → GIMP	0.103***	3.802	(0.064, 0.153)
*H6* MENG → CREL → ERES → GIMP	0.024***	2.726	(0.012, 0.040)

MENG: media engagement; CREL: cultural relatability; ERES: emotional resonance; GIMP: global impact; *** significance *p* < 0.05 (*t* > 1.96).

Table 6 presents the goodness-of-fit index (GFI) for the study’s constructs, providing an assessment of the measurement model’s validity and explanatory power. The average variance extracted (AVE) values demonstrate how well the items within each construct capture variance, with higher AVE values indicating stronger construct validity. The *R*^2^ values reflect the degree to which the proposed model explains variances in the constructs, underscoring the model’s predictive strength. The goodness-of-fit index (GFI) is calculated as the square root of the product of the average AVE and average *R*^2^, resulting in a GFI of 0.429. While this GFI value is slightly below conventional thresholds for a strong model fit, it still suggests that the model accounts for a reasonable portion of the variance in the constructs. The results highlight the model’s ability to capture the relationships between media engagement, cultural relatability, emotional resonance, global impact, and the moderating role of Chinese culture communication.

**Table 6:** Goodness-of-fit index (GFI).

Constructs	AVE	*R^2^*
MENG	0.649	
CREL	0.757	0.268
ERES	0.735	0.107
GIMP	0.673	0.461
CCC	0.503	
Average scores	0.663	0.278
(*GFI =* $\sqrt{\bar{\mathrm{AVE}}\times \bar{R^{2}}}$ )	0.429	

AVE: average variance extracted; MENG: media engagement; CREL: cultural relatability; ERES: emotional resonance; GIMP: global impact; CCC: Chinese culture communication.

## Discussion

The primary objective of this study is to examine the serial moderated mediation model that explores the relationships between media engagement, cultural relatability, emotional resonance, and global impact within the context of Chinese culture communication. Rather than merely confirming direct associations, the findings illuminate the sequential psychological processes through which engagement with cultural media content evolves into broader global cultural orientation. By situating media engagement within a structured cognitive–affective pathway (Petty et al., 1986; Zillmann, 2003), the study moves beyond surface-level media effects and clarifies how cultural narratives are internalized and transformed into attitudinal outcomes.

The first objective of evaluating the influence of media engagement on cultural relatability is achieved, as the findings indicate a significant positive relationship between these constructs. This outcome aligns with the work of Cheung et al. (2021), who emphasize that higher media engagement enhances audiences’ ability to relate to cultural content. Importantly, the findings extend prior work by demonstrating that engagement does not merely increase exposure or recall, but strengthens cognitive alignment with cultural narratives. This supports and deepens Cheung et al.’s (2021) argument by positioning cultural relatability as a structured evaluative mechanism rather than a peripheral outcome. Furthermore, the observed pathway from cultural relatability to emotional resonance corroborates Du et al. (2022), yet advances the literature by empirically embedding this relationship within a broader serial process. In turn, the positive influence of emotional resonance on global cultural orientation reinforces Huang et al. (2021), suggesting that emotional engagement serves as a catalyst for shaping broader intercultural perceptions rather than only immediate reactions.

The study also achieves its objective of exploring the mediating roles of cultural relatability and emotional resonance in the pathway from media engagement to global impact. The serial mediation analysis reveals that media engagement indirectly impacts global impact through both mediators, demonstrating a nuanced process where audiences first find the content relatable and then emotionally engaging before adopting broader cultural perspectives. This aligns with Bagga et al. (2023) and Priadi and Thariq (2023), who highlight the importance of mediation analysis in understanding complex relationships within cultural communication research.

Beyond methodological confirmation, this serial mediation finding provides theoretical support for the integrated ELM–ADT framework. Specifically, the results validate the proposition that central cognitive processing (ELM; Petty et al., 1986) precedes affective amplification (ADT; Zillmann, 2003), thereby offering empirical grounding for the cognitive–affective progression proposed in the theoretical background. By empirically demonstrating this layered structure, the study contributes to communication scholarship by showing that cultural influence unfolds through staged psychological mechanisms rather than isolated effects.

The moderating role of Chinese culture communication is also established, with results indicating that effective communication strategies strengthen the positive relationship between media engagement and cultural relatability. This finding supports Aririguzoh (2022) and Hodkinson (2024) perspective that authentic and clear cultural communication enhances the effectiveness of media content in bridging cultural understanding.

The moderation effect is particularly meaningful within the contemporary international communication environment, where geopolitical narratives and digital media ecosystems shape how cultural messages are received. In the context of China’s expanding global media presence and cultural diplomacy initiatives (Johnson et al., 2023; Gong et al., 2021), effective cultural articulation appears to function as a contextual amplifier, enhancing the cognitive accessibility and perceived authenticity of cultural narratives. This finding underlines that media engagement alone is insufficient; the quality and clarity of cultural communication determine whether engagement translates into meaningful cultural alignment.

Overall, the findings collectively demonstrate that global cultural orientation emerges from a structured interplay between engagement, cognitive alignment, affective resonance, and communication context. By situating these mechanisms within the broader landscape of cultural diplomacy and mediated globalization (Gerodimos, 2021; Kurki, 2022), the study offers both theoretical and practical contributions. Theoretically, it advances a unified cognitive–affective explanation of media-driven cultural influence. Practically, it suggests that policymakers and media strategists seeking to enhance China’s global cultural positioning should prioritize not only audience engagement but also culturally authentic and strategically articulated narratives.

Moreover, in an international environment characterized by heightened cultural competition, digital fragmentation, and shifting geopolitical perceptions, understanding how domestic audiences internalize globally oriented narratives becomes increasingly important. The findings indicate that shaping global cultural orientation begins with cultivating relatability and emotional resonance at the audience level, thereby reinforcing the strategic importance of coherent cultural communication in China’s evolving socio-political context.

### Theoretical implications

The theoretical implications of this study extend the existing body of knowledge on media engagement, cultural relatability, emotional resonance, global impact, and Chinese culture communication while contributing to the theoretical frameworks of the elaboration likelihood model (O’Keefe, 2013; Petty et al., 1986) and affective disposition theory (Zillmann, 2003; Zillmann and Cantor, 2017). Specifically, the study advances theoretical understanding by empirically validating a cognitive–affective sequence through which media engagement translates into global cultural orientation. By structuring these relationships within a serial moderated mediation framework, the findings clarify how cognitive evaluation (cultural relatability) and affective response (emotional resonance) jointly shape global influence. By integrating media engagement with cultural relatability and emotional resonance, the study not only reaffirms established relationships suggested by Lv et al. (2016) and Memon (2014) but also introduces the moderating role of Chinese culture communication, which has not been extensively explored in previous research.

This study makes a significant theoretical contribution to the elaboration likelihood model by demonstrating how media engagement operates as a central route of processing in enhancing cultural relatability. It extends ELM by showing that cultural relatability acts as a cognitive mediator that facilitates deeper audience engagement, leading to stronger emotional resonance and, ultimately, a global impact. In doing so, the study extends ELM beyond traditional persuasion settings by embedding it within a cross-cultural communication framework, demonstrating how central processing can generate culturally grounded evaluations rather than solely attitude change. This aligns with calls for more integrative communication models that capture layered media effects (Zhuang et al., 2023).

Additionally, the study provides empirical evidence to support the affective disposition theory, showing how positive emotional responses mediate the relationship between cultural relatability and global impact. By positioning emotional resonance as a key mediator, the study enhances ADT by illustrating how emotions serve as a bridge between cognitive evaluations (cultural relatability) and behavioral outcomes (global impact). Importantly, this contribution clarifies that emotional resonance is not merely an outcome of exposure but a structured affective mechanism that amplifies culturally grounded cognition into broader intercultural orientation.

Importantly, this contribution clarifies that emotional resonance is not merely an outcome of exposure but a structured affective mechanism that amplifies culturally grounded cognition into broader intercultural orientation. The findings suggest that Chinese culture communication strengthens the perceived relatability of media content and may indirectly support emotional resonance and global cultural orientation, offering practical implications for media producers and cultural policymakers seeking to refine communication strategies within an international context.

In terms of construct-specific contributions, this study expands the conceptual understanding of cultural relatability by demonstrating its dual role as a mediator and a foundational construct that enhances emotional engagement. It also redefines emotional resonance within the context of cultural communication, showing how emotions contribute to broader global impacts. The inclusion of Chinese culture communication as a moderator introduces a novel perspective to the literature, suggesting that cultural communication strategies play a pivotal role in shaping how media engagement translates into cultural influence.

Overall, the study integrates cognitive, affective, and contextual dimensions into a unified explanatory model of media-driven cultural influence. This framework contributes to international communication scholarship by clarifying how engagement-based persuasion evolves into global cultural orientation within a strategically mediated environment.

### Practical implications

The practical implications of this study provide valuable insights for media producers, cultural policymakers, and communication strategists seeking to enhance the global impact of media content, particularly within the context of Chinese culture communication. The findings highlight the importance of media engagement as a starting point for creating culturally relatable and emotionally resonant content. Media producers can leverage this insight by developing content that not only captures attention but also aligns with culturally diverse audience segments within an international communication framework. Rather than prioritizing surface-level visibility metrics, media organizations should strategically cultivate culturally grounded narratives that encourage deeper cognitive processing and emotional immersion. By focusing on cultural relatability, media creators can foster deeper connections with viewers, enhancing emotional resonance and increasing the likelihood of achieving a global impact. For instance, incorporating culturally relevant narratives, symbols, and values into media content can improve its effectiveness in promoting cultural understanding and appreciation amoniences.

Cultural policymakers, particularly those involved in promoting Chinese culture on the global stage, can apply these findings to optimize their cultural diplomacy and soft power strategies. The study reveals that effective Chinese culture communication strengthens the relationship between media engagement and cultural relatability, suggesting that strategic communication practices can amplify media influence. In the context of evolving geopolitical narratives and intensified global scrutiny of national image, coherent and authentic cultural messaging becomes increasingly critical.

Policymakers can design targeted initiatives that present Chinese culture authentically and clearly, helping to bridge cultural gaps and build a positive global image. Training programs for media professionals on effectively conveying Chinese cultural elements can be instrumental in achieving this objective. Additionally, collaboration with international media platforms to distribute culturally rich and emotionally engaging content can extend the reach and impact of Chinese culture globally.

For communication strategists, the study emphasizes the importance of managing both cognitive and emotional elements of media content. The serial mediation model suggests that content strategies should not only focus on relatability but also enhance the emotional journey of the audience. Strategic storytelling that integrates culturally meaningful symbols with emotionally compelling narratives may enhance both short-term engagement and long-term attitudinal alignment. These practical applications align with broader objectives of promoting intercultural dialogue and leveraging media as a tool for positive societal influence.

### Limitations

While this study provides significant insights into the relationships between media engagement, cultural relatability, emotional resonance, global impact, and Chinese culture communication, it is not without limitations. One limitation is the reliance on a convenience sample of university students, which may affect the generalizability of the findings to broader populations. Although university students are an appropriate group for understanding media engagement and cultural communication, future research could extend this study by including more diverse demographic groups, such as working professionals, international tourists, or media consumers from various cultural backgrounds. Additionally, while the study employs a time-lagged design, the intervals between data collection phases might not fully capture long-term changes in perceptions and attitudes. Longitudinal studies with extended time frames could provide deeper insights into how media exposure to cultural content influences global impact over time.

Future research could explore additional moderating and mediating variables to expand the current model’s explanatory power. For instance, individual differences such as cultural openness, media literacy, or prior exposure to Chinese culture could influence how media engagement translates into global impact. Researchers could also examine the role of different types of media (e.g., social media, films, documentaries) in enhancing cultural relatability and emotional resonance. Comparative studies involving different cultural contexts or examining cross-cultural interactions through media could further enrich the understanding of cultural communication strategies. Finally, experimental designs that manipulate media content and assess immediate and long-term audience reactions could offer robust evidence for causality, helping to validate and extend the theoretical frameworks of the elaboration likelihood model and affective disposition theory in the domain of international media studies.

## Conclusion

In conclusion, this study provides a comprehensive understanding of how media engagement influences global impact through the dual mediating roles of cultural relatability and emotional resonance, with Chinese culture communication acting as a critical moderator. By integrating the elaboration likelihood model and affective disposition theory, the study offers both theoretical and practical insights into the nuanced processes through which media content can shape global cultural perceptions. The findings demonstrate that effective media strategies, particularly those incorporating clear and authentic Chinese cultural communication, can significantly enhance cultural exchange and promote a positive global image. While acknowledging its limitations, the study opens new avenues for future research, contributing to the growing field of international communication and cultural diplomacy and offering actionable guidance for media producers, policymakers, and communication strategists aiming to optimize the influence of cultural media on global audiences.

## Data Availability

The raw data supporting the conclusions of this article will be made available by the authors, without undue reservation.

## Appendix 1: Questionnaire items

### Media engagement

1. I often find myself deeply engaged when interacting with media content related to Chinese culture.
2. I actively participate and respond to media content about Chinese culture.
3. Media content related to Chinese culture captures my attention effectively.
4. I feel emotionally involved while consuming media content about Chinese culture.

### Cultural relatability

1. I find the media content related to Chinese culture reflects my own cultural experiences.
2. The media content presents cultural values that I can easily relate to.
3. I feel a sense of familiarity with the cultural elements presented in the media content.

### Emotional resonance

1. The media content about Chinese culture evokes strong emotions in me.
2. I feel a deep emotional connection to the stories and messages conveyed through the media content.
3. The media content leaves a lasting emotional impression on me.

### Global impact

1. Media content about Chinese culture influences my perspective on global cultural diversity.
2. I am more open to learning about different cultures after engaging with Chinese media content.
3. The media content enhances my understanding of Chinese culture in a global context.

### Chinese culture communication

1. The media content effectively communicates Chinese cultural values.
2. I find the messages about Chinese culture to be clear and understandable.
3. The media content presents an authentic view of Chinese culture.
4. The media content provides insightful information about Chinese traditions and customs.
5. I believe the media accurately portrays the diversity of Chinese culture.
6. The communication style of Chinese culture in the media is engaging.
7. The media content strengthens my interest in Chinese culture.
