# Correction: From media engagement to global impact: a serial moderated mediation model of cultural relatability and emotional resonance in Chinese culture communication

**DOI:** 10.3389/fpsyg.2026.1850376

**Published:** 2026-04-21

**Authors:** Mengying Xiong, Yijin Chen, Yunqian Zhou

**Affiliations:** 1 School of Journalism and Communication, Nanchang University, Nanchang, China; 2 School of Broadcasting and Hosting Art, Wuhan College of Communication, Wuhan, China

**Keywords:** affective disposition theory, Chinese culture communication, cultural diplomacy, cultural relatability, elaboration likelihood model, emotional resonance, global impact, media engagement

In the published article, affiliation [Wuhan College of Communication, Wuhan] was erroneously given as [Wuhan University of Communication, Wuhan].

The original version of this article has been updated.

